# Prediction Model for the Carbonation of Post-Repair Materials in Carbonated RC Structures

**DOI:** 10.3390/ma10050492

**Published:** 2017-05-03

**Authors:** Hyung-Min Lee, Han-Seung Lee, Jitendra Kumar Singh

**Affiliations:** Department of Architectural Engineering, Hanyang University, 1271 Sa 3-dong, Sangrok-gu, Ansan 15588, Korea; abmcl@hanyang.ac.kr (H.-M.L.); jk200386@hanyang.ac.kr (J.K.S.)

**Keywords:** carbonation, repair, prediction, post repair, carbonation depth, FEM analysis, FDM analysis

## Abstract

Concrete carbonation damages the passive film that surrounds reinforcement bars, resulting in their exposure to corrosion. Studies on the prediction of concrete carbonation are thus of great significance. The repair of pre-built reinforced concrete (RC) structures by methods such as remodeling was recently introduced. While many studies have been conducted on the progress of carbonation in newly constructed buildings and RC structures fitted with new repair materials, the prediction of post-repair carbonation has not been considered. In the present study, accelerated carbonation was carried out to investigate RC structures following surface layer repair, in order to determine the carbonation depth. To validate the obtained results, a second experiment was performed under the same conditions to determine the carbonation depth by the Finite Difference Method (FDM) and Finite Element Method (FEM). For the accelerated carbonation experiment, FDM and FEM analyses, produced very similar results, thus confirming that the carbonation depth in an RC structure after surface layer repair can be predicted with accuracy. The specimen repaired using inhibiting surface coating (ISC) had the highest carbonation penetration of 19.81, while this value was the lowest for the corrosion inhibiting mortar (IM) with 13.39 mm. In addition, the carbonation depth predicted by using the carbonation prediction formula after repair indicated that that the analytical and experimental values are almost identical if the initial concentration of Ca(OH)_2_ is assumed to be 52%.

## 1. Introduction

Concrete carbonation is one of the main causes for the deterioration of reinforced concrete (RC) structures. This phenomenon is caused by the penetration of atmospheric CO_2_ into the concrete, resulting in decreased basicity [1,2,3,4,5,6]. Concrete carbonation is a complex physicochemical process that takes place upon prolonged exposure to atmospheric CO_2_. The concentration of CO_2_ is variable and depends on the different climatic and atmospheric conditions. Generally, 0.03% CO_2_ is present in normal air while in industrially polluted environment, it could reach around 0.3% and at such concentrations, CO_2_ can penetrate easily if there is no protection applied on the concrete cover [7,8]. This process destroys the passive film that surrounds the reinforcement bars exposing them to corrosive attack [9,10,11,12,13,14].

The carbonation of concrete is one of the most disruptive processes that affects the durability of concrete and causes a significant reduction in its service life [15,16].

Reinforcement bars can be inflated due to corrosion, which causes the concrete to crack, resulting in decreased strength of the member and degradation of its structural durability. This problem has necessitated the repair of many RC structures in recent times. However, there has been almost no study on post-repair concrete carbonation. Many researchers have proposed carbonation estimation equations, which enable the prediction of the carbonation depth. Such equations were based on results obtained under conditions of indoor accelerated carbonation and exposure experiments, which were performed on concrete that had not undergone repair. Such tests have also been used to develop equations to evaluate the carbonation depth from which the endurance life of concrete can be predicted.

The reduction in durability of RC structures can be caused by a destruction of concrete or the corrosion of the reinforcement bars or a combination of both [17].

In the report by RILEM, it was stated that a high amount of CH and CSH in concrete might reduce the penetration of CO_2_ [18], but until now it is a controversial topic. It has also been reported that both the type of filler and the cement paste composition play major roles.

A prognosis durability model has been discussed by Czarnecki and Woyciechowski (2013) for the repair of RC structures that suffered carbonation and chloride attack [19].

Neves et al. [20] examined the carbonation resistance of concrete by measuring its gas permeability. Kuosa et al. [21] presented an equation to predict the carbonation caused only by freezing and thawing, from which the endurance life of the concrete could be evaluated. Duprat et al. [22] conducted accelerated carbonation tests on unrepaired concretes for a probabilistic durability prediction. Kashef-Haghighi et al. [23] developed a mathematical model of CO_2_ absorption based on an accelerated carbonation test on concrete without repair materials. Silva et al. [24] investigated the carbonation process in recycled aggregate concretes, while Hills et al. [25] statistically analyzed the carbonation rate in concretes containing no repair materials. Also, the Korea Concrete Institute (KCI) [26] predicted the rate of carbonation as a function of the water/binder (W/B) ratio.

While the previous studies mentioned above have predicted the carbonation of only concrete without considering repair materials, there have been several other works which have also taken repair materials into consideration in their studies. The accelerated carbonation test for the durability of concrete was mentioned in the Chinese National Standard GB/T 50082-2009 titled “Standard for test methods of long-term performance and durability of ordinary concrete” [27]. The Architectural Institute of Japan (AIJ) [28] also assigned coefficients to different types of repair materials for the prediction of carbonation. In addition, Köliö et al. [29] investigated the corrosion of a reinforcement bar induced by the carbonation of a concrete repair material additive. However, the carbonation prediction equations derived in these studies only considered the carbonation in the original concrete, or carbonation in a concrete to which a repair material had already been applied.

Liu et al. have proposed a mechanism to understand the effect of carbonation and chloride aerosol attack in ordinary Portland cement concrete using 20% CO_2_ [30]. On the other hand, Mohammed et al have investigated the 100% carbonation of self-compacting concrete and found that the replacement of limestone powder (LP) increased the depth of carbonation during the accelerated test, whereas there was no effect if fly ash (FA) or a combination of fly ash and silica fume (FA + SF) was used [31]. Since there are only a few reports on the accelerated carbonation in post-repair carbonated concrete, especially with respect to experimental and analytical data, there is a need to investigate this phenomenon. Therefore, in the present study, we have focused on concrete structures in which carbonation was already underway. Carbonated building structures were simulated by accelerated carbonation as a sequel to surface repair. The carbonation coefficients for different repair materials were compared by conducting accelerated carbonation tests using 20% CO_2_ [31,32]. From the values of carbonation coefficients obtained for the different repair materials, the progress of carbonation was predicted using the prediction equation for post-repair carbonation. The reliability of the predicted carbonation depths was examined using both the Finite Difference Method (FDM) and the Finite Element Method (FEM).

## 2. Experimental

### 2.1. Experimental Design

**Figure 1:**
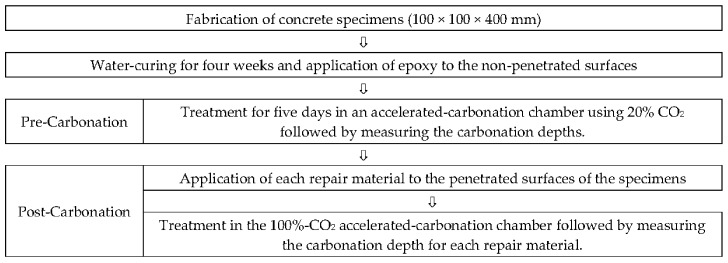
Flow chart of the experimental procedure.

The experiments were grouped according to the nature of the repair materials present; each set of sample was tested in triplicate. The considered repair materials were Organic Alkaline Inhibitor (OAI), Inhibiting Surface Coating (ISC), Corrosion-Inhibiting Mortar (IM), and Water-Based Paint (WP). Table 1 lists the concrete compositions. The concrete specimens were fabricated with a water/cement ratio of 55% using a 100 × 100 × 400 mm^3^ concrete mold. As described in Figure 1, after curing the specimens in water for 28 days, epoxy was applied to the non-penetrated surface. The specimens were subsequently pre-carbonated for five days in a 20%-CO_2_ accelerated-carbonation chamber (20 °C and 60% Relative Humidity) and the carbonation depths were measured by the KS F 2596 method [33]. Each repair material was then applied to the penetrated surfaces of the specimens, which were treated again in the 20%-CO_2_ accelerated-carbonation chamber. The carbonation depths were measured again.

### 2.2. Selected Materials

Type 1 general-purpose Portland cement was used. Table 2 gives the physical properties of the employed OAI, the main ingredient of which was (CH_3_)_2_NCH_2_CH_2_OH with a pH of 11.9, and specific gravity of 1.07. The ISC material contained latex with a viscosity of <20 centipoise (CPS). The IM had a solid acrylic polymer content of >98% and a pH in the range 6.5–8.5. The WP used was mainly composed of an acrylic emulsion resin and had a density of 1.63 g/cm^3^.

### 2.3. Fabrication of Specimens and Experimental Method

**Figure 2:**
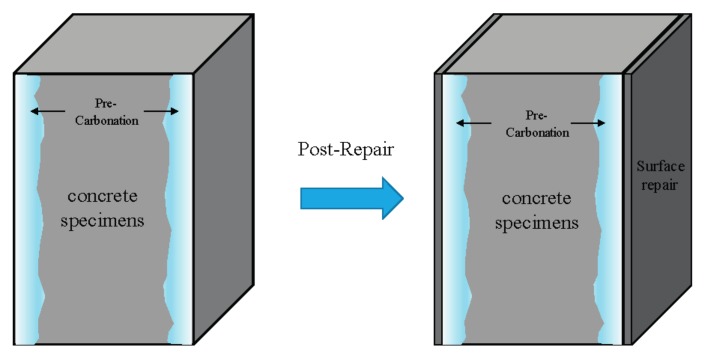
A description of the process of application of repair materials to concrete specimens.

Figure 2 describes the application of the repair materials to the concrete specimens. In the experiments to predict concrete carbonation after surface repair, epoxy was applied to the surfaces that were not penetrated by CO_2_. The pre-carbonation was performed for five days in a 20% CO_2_ accelerated-carbonation chamber, after which the specimens were split to measure the carbonation depth. The same experiment was repeated by applying other repair materials on the surfaces of the concrete specimens after pre-carbonation and the corresponding carbonation depths were measured. The thickness of the repair materials WP and IM were measured by a non-destructive technique using Elcometer 456 (Elcometer, Tokyo, Japan), while for OAI and ISC, this was carried out using a caliper at different places on the concrete surface. Table 3 gives the thickness of each repair material that was applied. IM exhibited higher thickness while OAI spread all over the surface through the pores in the concrete. OAI is a liquid while ISC is a viscous material. Therefore, it is noted here that the thicknesses of the different applied repair materials are different.

## 3. Experimental Results and Analysis

### 3.1. Carbonation Depth and Rate after Repair

#### 3.1.1. Carbonation Depth after Repair

**Figure 3:**
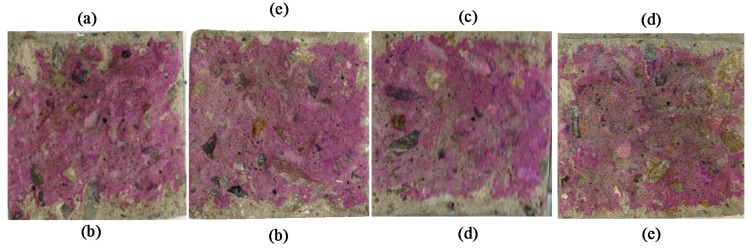
Measured carbonation depths of the split specimens. (**a**) Unrepaired, (**b**) Water-Based Paint (WP), (**c**) Organic Alkaline Inhibitor (OAI), (**d**) Inhibiting Surface Coating (ISC) and (**e**) Corrosion-Inhibiting Mortar (IM).

Figure 3 shows the physical carbonation depths of split specimens. The measurement was done in accordance with KS F 2596 [20] after the repair materials had been left to dry for 7 days. This figure clearly shows that after spraying of phenolphthalein indicator on specimens, IM exhibited minimum spreading. This result indicates that IM repair material has minimum carbonation. A second carbonation process was then performed on the specimens in the 20% CO_2_ carbonation chamber under the same conditions as the pre-carbonation. The carbonation depths were then measured again for 7 and 28 days after the initiation of the carbonation process.

**Figure 4:**
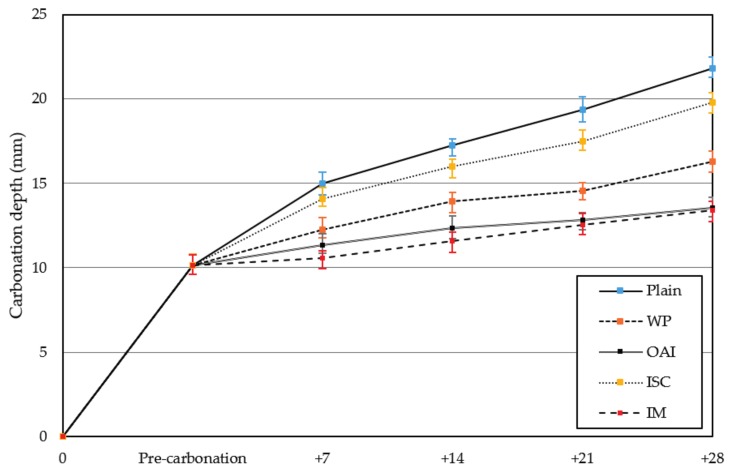
Carbonation depths after accelerated carbonation.

Figure 4 also shows the measured carbonation depths for 7 days after the initiation of accelerated carbonation following the application of the repair materials and error bar for corresponding specimens. It can be observed that the unrepaired specimen has a carbonation depth of about 14.98 mm, which is 4.85 mm greater than that after pre-carbonation. This figure shows the carbonation depth after the application of the repair materials as well as that obtained under accelerated conditions. Comparison of the carbonation depths after 28 days for the different repair materials reveals that the value for ISC is the highest (~19.81 mm), while that for IM is the lowest (13.39 mm). It is may be due to that IM contain acrylic polymer that resist the penetration of CO_2_ toward concrete and work as barrier.

#### 3.1.2. Carbonation Rate

To compare the concrete carbonation rates for the different repair materials, it is necessary to correct for the 20% CO_2_ concentration during accelerated carbonation test, so that the results will be applicable to normal atmospheric conditions. The carbonation coefficient was calculated using Equation (1) [24]. The results are given in Table 4.

(1) $$C=A\sqrt{CO_{2}/0.05}\times \sqrt{t}$$

where *C* is the carbonation depth (mm), *t* is the time (year), *A* is the carbonation coefficient ($\mathrm{mm}/\sqrt{year}$) and *CO*_2_ is the *CO*_2_ concentration in the atmosphere (%).

As can be observed from above table, the carbonation coefficient is different for the various specimens tested. The Unrepaired specimen shows a value of 9.4 $\mathrm{mm}/\sqrt{year}$, while the specimen repaired by IM has a low value of only 1.5 $\mathrm{mm}/\sqrt{year}$.

The carbonation rate for each repair material can be determined using Equation (2). The carbonation rate is the ratio of the carbonation coefficient of the specimen using a given repair material ($A^{\prime }$) to that of the unrepaired specimen (*A*), which is assumed to be 100%. The carbonation rate can be assessed based on Equation (2).

(2) $$\text{Carbonation Rate}=\frac{A^{\prime }}{A}\times 100$$

where $A^{\prime }$ is the carbonation coefficient of the specimen with a given repair material ($\mathrm{mm}/\sqrt{year}$), and *A* is the carbonation coefficient of the unrepaired specimen ($\mathrm{mm}/\sqrt{year}$).

**Figure 5:**
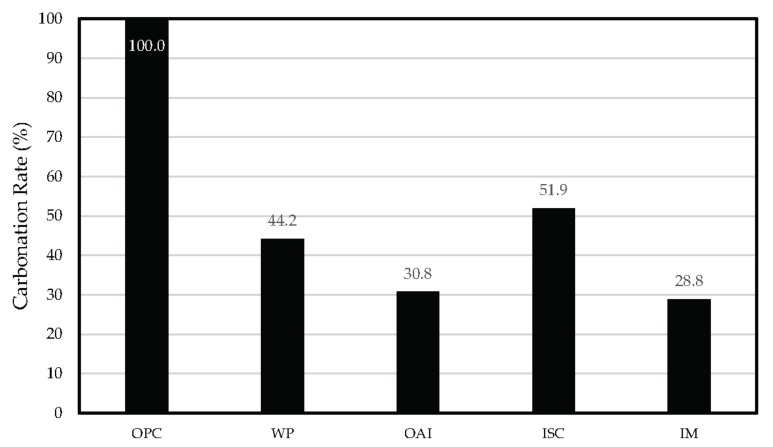
Comparison of the carbonation rates for the different specimens with ordinary Portland cement (OPC).

Figure 5 shows the comparison of the carbonation rates of the different specimens. The specimen repaired with ISC has the highest carbonation rate, which implies that it has the lowest carbonation resistance. The specimens repaired with WP and OAI have carbonation rates of 44.2% and 30.8%, respectively, with the IM-repaired specimen having the lowest value of 28.8%, pointing to the best carbonation resistance. This result is attributed to the fact that moisture permeation is blocked by the acrylic polymer which was tightly bound to the interface of the concrete.

### 3.2. Prediction of the Progress of Post-Repair Carbonation Depth by Accelerated Carbonation Test 

**Figure 6:**
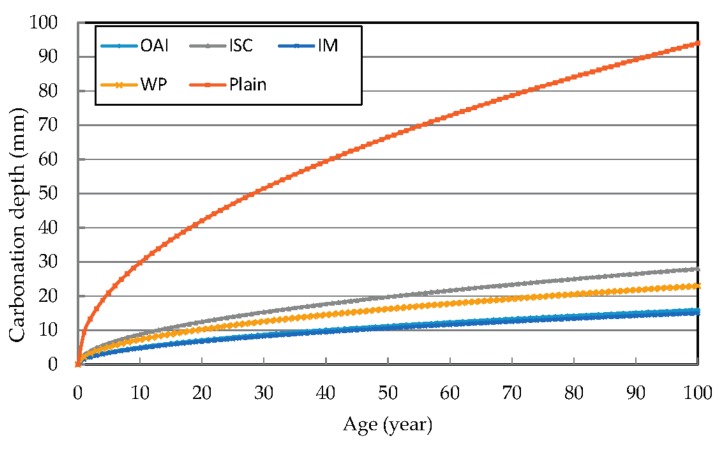
Predicted progression of the carbonation depth for the different repair materials.

Figure 6 compares the predicted progression of the carbonation depth for the different repair materials up to 100 years of age. The carbonation depth after 10 years of repair by IM is 4.74 mm, from which it can be deduced that the repair affords a carbonation resistance effect of about 25.26 mm compared to the unrepaired sample. Table 5 compares the carbonation depths for the different repair materials.

A comparison of the carbonation depth values revealed that it is up to 30 mm for the unrepaired specimen after 10 years, which is close to the thickness of the concrete surface layering in the unrepaired specimen. The predicted progression of the carbonation depth in each of the specimens containing repair materials was around 10 mm or lower.

To compare the predicted post-repair progression of carbonation for different repair materials, the carbonation coefficients previously obtained for the repair materials were plugged in. The equations used to predict the progress of the respective post-repair carbonation process are presented in Table 6.

In the case of the unrepaired specimen, the equation to predict the geometric carbonation progress can be derived to be $C=9.4\sqrt{t_{0}}$, while the equation for the case using the repair material WP is $C^{\prime }=2.3\left(\sqrt{t}-\sqrt{t_{0}}\right)+9.4\sqrt{t_{0}}$.

**Figure 7:**
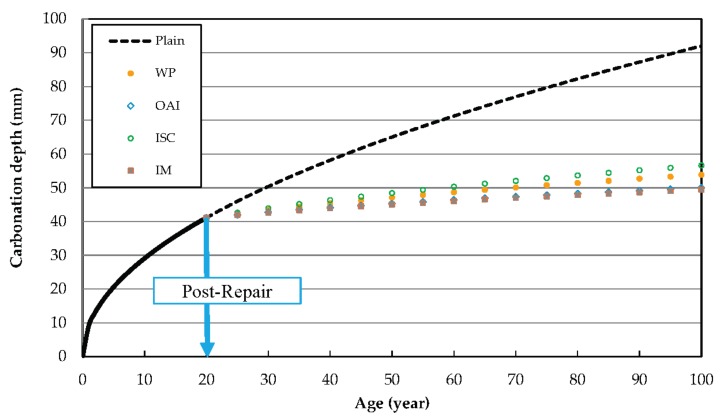
Predicted post-repair progress of carbonation using different repair materials.

Figure 7 shows the predicted post-repair progression of carbonation. The plots are graphical representations of the carbonation progress prediction equations shown in Table 6. As can be observed, the sample repaired using ISC shows 56.6 mm penetration over 100 years, while using WP results in a carbonation penetration of 53.8 mm in just 20 years. Moreover, 20 years after repair with IM, the carbonation resistance effect is ~42.5 mm relative to the unrepaired sample.

### 3.3. Prediction of Post-Repair Carbonation by Finite Element Method (FEM) and Finite Difference Method (FDM)

#### 3.3.1. FEM Analysis of Post-Repair Carbonation Depth

FEM analysis was performed using the LECCA2 program from the Architectural Institute of Japan to predict the depth of carbonation by considering the applied repair materials [28]. The input conditions for the analysis include a temperature of 20 °C with 60% humidity as indicated in Table 7.

The cover thickness of the reinforcement bar was assumed to be 30 mm and it was also assumed that the fundamental material properties of concrete were applicable to the fabricated specimens. Equations (3) and (4), which are the carbonation prediction equations provided by AIJ, were used for the FEM analysis [28].

(3) $$C=A\left(\sqrt{\left(t+{R_{1}}^{2}\right)}-R_{2}\right)$$

(4) $$A=k\times \alpha_{1}\times \alpha_{2}\times \alpha_{3}\times \beta_{1}\times \beta_{2}\times \beta_{3}$$

Here, C is the carbonation depth (mm), *t* is the time (year), $R_{1}$ is the coefficient of Repair 1, $R_{2}$ is the coefficient of Repair 2, α_1_ is the coefficient of concrete type, α_2_ is the coefficient of cement type, α_3_ is the coefficient of W/C, β_1_ is the temperature, β_2_ represents the humidity, β_3_ is the CO_2_ concentration and *k* is the coefficient of Kisitani Equation or Shirayama Equation.

**Figure 8:**
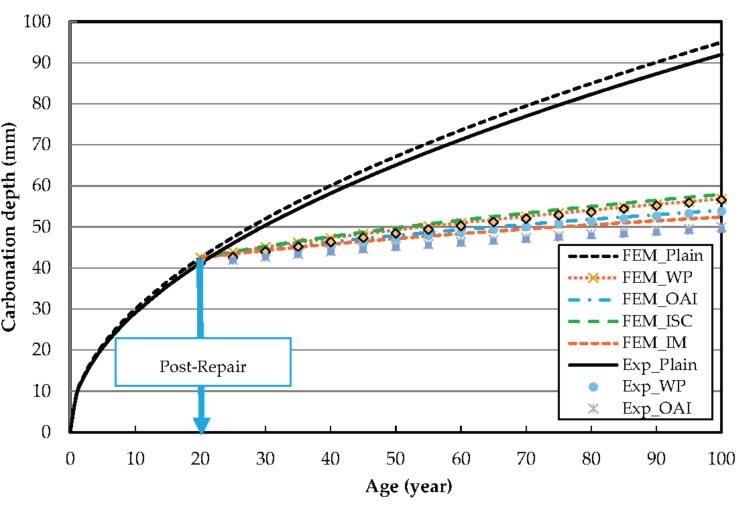
Carbonation progress predicted by FEM analysis.

Figure 8 compares the carbonation depth values predicted by FEM analysis with the results of the accelerated carbonation tests. The FEM analysis gives information on the prediction of carbonation of repair materials exposed to accelerated carbonation with 20% CO_2_. It can be observed that the penetration in the unrepaired specimen after 100 years as determined by FEM analysis is only 3 mm higher than the experimentally determined value. Figure 8 shows the excellent fit of the values of carbonation depth at longer durations of exposure predicted by the model with our experimental results.

Similarly, differences of 3, 4.11, 1.34, and 3 mm can be observed for the repair materials of WP, OAI, ISC, and IM, respectively. These differences are attributed to various variables, such as the diffusion of Ca(OH)_2_ at the interface between the concrete and the finish coat.

#### 3.3.2. FDM Analysis of the Post-Repair Carbonation Depth 

**Figure 9:**
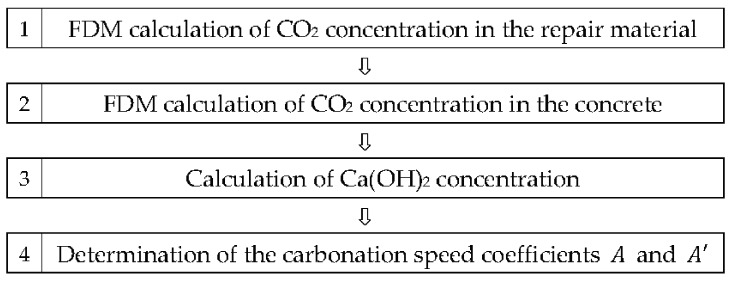
Flowchart of the FDM analysis.

Figure 9 shows the flowchart of the FDM analysis. The movement of CO_2_ in concrete was set to occur in one direction but the movement of Ca(OH)_2_ in the concrete was not considered. Table 8 and Table 9 specify the input variables for the concrete and repair materials, respectively [34].

The FDM analysis was conducted using various input conditions and Equation (5). It was assumed that the repair was necessitated by carbonation-induced corrosion of the reinforcement bars when the Ca(OH)_2_ concentration below a depth of 20 mm was reduced to 40–70% of the initial value [35].

(5) $$P=D_{CO_{2}}\times S$$

where *Dco*_2_ is the diffusion coefficient of CO_2_ (m^2^/s), *P* is the air permeability (cm^3^ (STP) × cm/(cm^2^ × s × cm Hg)), and *S* is the solubility coefficient (mol/m^3^ × Pa).

**Figure 10:**
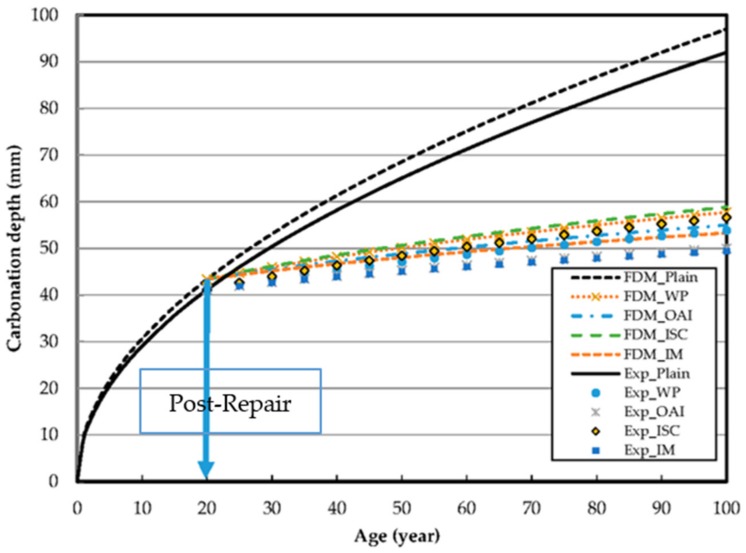
Carbonation progress predicted from FDM analysis.

Figure 10 shows the distribution of Ca(OH)_2_ concentration after 20 years of repair. It is assumed that the initial concentration of Ca(OH)_2_ is 40% at the time of the repair using different repair materials. The predicted values for the progress of carbonation with exposure time using the FDM model agree very well with our experimental data.

## 4. Conclusions

In this study, the prediction of the progress of carbonation in RC structures with the repair materials following a previous carbonation process was investigated by conducting accelerated carbonation tests combined with FEM and FDM analyses. A summary of our findings is as follows:

1. A comparison of the experimentally determined carbonation depths in specimens containing different repair materials revealed that the specimen repaired using ISC had the highest carbonation penetration of 19.81 mm, while that with IM had the lowest carbonation penetration of 13.39 mm. This result implies that the latter exhibited the best carbonation penetration resistance and is, consequently, the best repair material among those chosen in this study.
2. The comparison of the experimental values with and those predicted from FDM analysis are in agreement, and show identical results at 52% Ca(OH)_2_.
3. The FEM and FDM results confirmed the feasibility of predicting the carbonation rate of RC structures through accelerated tests. Its purpose of assessing the endurance life of such structures was fulfilled. Both techniques confirmed IM to be the best material to provide carbonation resistance to concrete under an atmosphere containing 20% CO_2_.

## Figures and Tables

**Table 1:** Concrete mix proportions. W/C: Water/Cement.

W/C	Unit Weight (kg/m^3^)			
	Water	Cement	Sand	Coarse Aggregates
0.55	173	315	891	923

**Table 2:** Physical properties of the Organic Alkaline Inhibitor (OAI).

Type	Specific Gravity	pH	Viscosity(CPS)	Main Component
Organic inhibitor	1.07	11.9	11	(CH_3_)_2_NCH_2_CH_2_OH

**Table 3:** Thickness of each repair material.

Sl. No.	Type	Thickness (mm)
1	WP	0.1
2	OAI	spread
3	ISC	2
4	IM	6

WP: Water-Based Paint; ISC: Inhibiting Surface Coating; IM: Corrosion-Inhibiting Mortar.

**Table 4:** Carbonation coefficients under normal atmospheric conditions.

Sl. No.	Type of Concrete Specimens	Carbonation Coefficient ($\mathrm{mm}/\sqrt{year}$)
1	Unrepaired	9.4
2	WP	2.3
3	OAI	1.6
4	ISC	2.8
5	IM	1.5

**Table 5:** Comparison of the carbonation depths for the different repair materials. (After 10 years)

Sl. No.	Type of Concrete Specimen	Carbonation Depth (mm)
1	Unrepaired	30
2	WP	7.27
3	OAI	5.06
4	ISC	8.85
5	IM	4.74

**Table 6:** Equations to predict progress of carbonation.

Type of Concrete Specimen	Carbonation Progress Prediction Equation
Unrepair	$C=9.4\sqrt{t_{0}}$
WP	$C=2.3\left(\sqrt{t}-\sqrt{t_{0}}\right)+9.4\sqrt{t_{0}}$
OAI	$C=1.6\left(\sqrt{t}-\sqrt{t_{0}}\right)+9.4\sqrt{t_{0}}$
ISC	$C=2.8\left(\sqrt{t}-\sqrt{t_{0}}\right)+9.4\sqrt{t_{0}}$
IM	$C=1.5\left(\sqrt{t}-\sqrt{t_{0}}\right)+9.4\sqrt{t_{0}}$

C is the carbonation depth after repair (mm), $t_{0}$ is the time of repair (year), and *t* is the time (year).

**Table 7:** Input data for Finite Element Method (FEM) analysis.

Description	Input Parameter	Unit
Cover thickness of the reinforcement bar	30	mm
α_1_ (Coefficient of concrete type)	1	-
α_2_ (Coefficient of cement type)	1	-
α_3_ (W/C coefficient)	0.17	-
β_1_ (temperature)	20	°C
β_2_ (Humidity)	60	%
β_3_ (CO_2_ Concentration)	0.05	%
*k* (coefficient of Kisitani Equation or Shirayama Equation)	1.72	-
*R*_1_ (Repair coefficient 1)	0	-
*R*_2_ (Repair coefficient 2)	30	-
Initial Ca(OH)_2_ Concentration	0.876	mol/cm^3^
Reaction rate constant	500,000	1/day
Ca(OH)_2_ loss	40	%

**Table 8:** Input variables for concrete.

Parameter	Input Value	Unit
Diffusion coefficient of CO_2_ in concrete	0.0005	cm^2^/day
Rate constant (k) of the reaction between CO_2_ and Ca(OH)_2_	500,000	1/day
Thickness of the concrete specimen	20	cm
Ca(OH)_2_ concentration on the specimen surface	0.000003	mol/cm^3^

**Table 9:** Input variables for the repair materials.

Type	Diffusion Coefficient of CO_2_ (m^2^/s)	Air Permeability (cm^3^ (STP) × cm/(cm^2^ × s × cm Hg))
WP	0.000055	0.06
OAI	0.000052	1
ISC	0.000058	0.2
IM	0.000018	0.2
